# Profiling of antibiotic resistance among uropathogens isolated from patients attending Kericho County Referral Hospital

**DOI:** 10.11604/pamj.2023.45.19.19585

**Published:** 2023-05-05

**Authors:** Gladys Chepkoech Mosonik, Janeth Jemutai Kombich

**Affiliations:** 1 Department of Biological Sciences, University of Kabianga, Kericho, Kenya

**Keywords:** Uropathogens, antibiotic resistance, *Staphylococcus aureus*, *E. coli*

## Abstract

**Introduction:**

urinary tract infection (UTI) comes second after respiratory infections in most communities and hospital settings, affecting people of all ages. Frequent use of antibiotics to manage UTI has resulted in development of resistance, calling upon policymakers to fast-track and enforce policies that guide the use of antibiotics in the country. This study intended to determine the current antibiotic resistance to uropathogens among patients attending Kericho County Referral Hospital.

**Methods:**

three hundred urine samples from eligible participants were cultured and bacteria colonies identified using biochemical tests. Antibiotic sensitivity was done using Kirby Bauer disk diffusion method on Mueller Hinton Agar. **Results:** the aetiological agents of UTI were Staphylococcus aureus, Enterococci faecalis, E. coli, Proteus spp and Klebsiella pneumonia. Antibiotic resistance was observed among these uropathogens to commonly used antibiotics namely; ampicillin (84.3%), azithromycin (71.9%) and augmentin (69.8%). However, there were some bacteria that were susceptible to all or some commonly used antibiotics. There was moderate resistance to norfloxacin (43%) except in Staphylococcus aureus which showed 64% resistance. The isolates showed less resistance to cefoxitine (13.2%), gentamycin (11.6%) and ciprofloxacin (10%). While most bacteria showed multiple resistance to 3 drugs, some showed resistance to at most 5 drugs tested in the study. **Conclusion:** this study found Staphylococcus aureus to be the predominant aetiological agent of UTI. Cefoxitine, gentamycin and ciprofloxacin are good therapeutic choices for confirmed recurrent UTI when culture results are unavailable. There is need to have regular screening of aetiological agents of UTI and their resistance to antimicrobials.

## Introduction

Urinary tract infections come second after respiratory infections in most communities and hospital settings. Recurrence of the infection, especially in vesico-ureteral reflux, may lead to long-term sequelae such as end-stage renal disease and renal scar [1]. It is a common infection in women and a major health problem reported in 20% of pregnant women [2]. Antibiotics are often used to prevent recurrence and subsequent permanent renal damage. Gram-negative bacteria, particularly *Escherichia coli* colonizes the intestines a few hours after birth. Although it forms part of the normal flora, it is also considered a primary uropathogen [3]. On the other hand, literature strongly suggests that gram-positive uropathogens may be easily overlooked due to limited culture based assays often used in many hospital diagnostic laboratories [4,5]. Treatment of UTI is often given empirically and is based on information determined from antibiotic resistance pattern. Effective antibiotic treatment is essential as a preventive and curative measure, protecting patients from potentially fatal diseases. However, increase in uncontrolled usage of antibiotics has led to an emergence of antibiotic resistance [6] which complicates antibiotic therapy. Multinational organisations such as US Centre for Disease Control (CDC), World Health Organisation (WHO), and European Centre for Disease Prevention and Control (ECDC) have documented the diseases caused by multidrug resistance organisms as a global threat to public health [7,8].

Emergence of this antimicrobial resistance has been attributed to non-compliance to drug prescription [9] over-the-counter prescription and presence of substandard drugs in the market [10]. Antibiotic resistance in most bacteria, in particular those belonging to the family *Enterobacteriaceae- E. coli* and *Klebsiella pneumonia*, have led to recurrence of the infection, hence resulting in ineffective management of UTI [11]. The European Centre for Disease Prevention and Control has also reported a widespread increase in antibiotic resistance of *E. coli* and *K. pneumonia* to carbapenems and multi-drug resistant *Staphylococcus aureus* [12]. Epidemiological studies conducted in different regions of the world indicate that multi-drug resistant bacteria are responsible for 23-51% of community/hospital acquired infections, which include but not limited to urinary tract infection [13-15]. Epidemiological and antibiotic resistance patterns of uropathogens vary inter-regionally. The patterns of antibiotic resistance are continually changing due to different antibiotic treatment regime [16]. Susceptibility of bacterial pathogens to antimicrobials also varies regionally in any country [17]. This is because in most cases of UTIs empirical treatment is initiated before culture results are observed and thus antimicrobial resistance to uropathogens might increase due to inappropriate antibiotic choice. The World Health Organisation and European Union have recognized the importance of studying the trends of antimicrobial resistance and the strategic development to combat it since it is a public health concern [18]. The regional study of antibiotic resistance patterns and its epidemiology is therefore critical in providing the surveillance data to clinicians to enable them to make the right choices when managing patients.

**General objective:** the main objective of this study was to determine the antibiotic resistance pattern of uropathogens causing UTI among patients attending Kericho County Referral Hospital (KCRH).

**Specific objectives:** the specific objectives of the study were to: determine the demographic characteristics of patients with UTI attending KCRH; identify uropathogens in urine samples from patients with UTI attending KCRH; determine the antibiotic resistance profiles of uropathogens isolated from patients with UTI attending KCRH.

**Hypotheses of the study:** there is no statistically significant antibiotic resistance in bacteria isolated in urine samples from patients with UTI in KCRH.

## Methods

**Study design:** this was a hospital based cross-sectional study and eligible participants were outpatients clinically diagnosed with urinary tract infection in the routine clinical workflow and who consented to participate in the study.

**Setting:** the study was conducted at Kericho County Referral Hospital (KCRH) laboratory from January to May 2018. Kericho County Referral Hospital is located in Kericho town. Approximately 9,000 outpatients and 1,350 in-patients attend KCRH per month as per the Ministry of Health report of 2018; it has a bed capacity of 250.

**Participants:** sample size calculation was determined using Fisher´s formula for estimating minimum sample size for prevalence studies [19] using 20.6% prevalence [20]. Three hundred participants were recruited in the study. The study population included all out-patients who sought treatment at KCRH and consented to participate in the study.

**Inclusion criteria:** patients with UTI who gave written consent or assent. Patients who were already diagnosed with UTI and have been on antibiotic therapy within the past two weeks were excluded in the study.

**Variables:** the variables that were the determinants of the main hypothesis were the identified organism and the susceptibility of the organism to the antibiotics in the study. Demographic data were obtained through a structured questionnaire interview.

**Data sources:** the data for the above variables were obtained using the following procedures.

**Urine sample collection and analysis:** the participants were asked to aseptically collect clean catch midstream urine samples and avail to the lab immediately. Samples were first cultured and then examined macroscopically for physical characteristics such as colour and turbidity. Microscopically, samples were observed for the presence of deposits. Dipstick screening was done for biochemical test of leukocytes, nitrite, Ph, specific gravity, bilirubin, protein, glucose, urobilinogen, ketones and presence of blood.

**Bacterial culture:** ten microlitres (μl) of the urine sample were cultured asceptically onto blood agar base agar media with 5% sheep blood, MacConkey agar and Cystein Lactose Electrolyte Deficient (CLED) agar media (oxoid Ltd, Basingstoke, UK) using a standard bacteriological loop by streaking and incubated aerobically at 35^0^C for 24 hours. Plates with pure colony count of ≥104 CFU were considered significant bacteriuria. Bacterial colony identification was done based on colony morphology, gram stain and standard biochemical tests. Gram-positive bacteria identification was done using catalase test, coagulase test and API test. Biochemical tests for Gram-negative bacteria isolates were Tripple Sugar Iron, Simmon´s citrate agar, Vorges-Proskauer, motility-indole-urease, catalase, oxidase and API 20 method Beckton Dickson USA [21,22].

**Antibiotic susceptibility testing:** antibiotic susceptibility test was done for bacterial isolates using Kirby Bauer disk diffusion method [23] following the guidelines recommended by Clinical Laboratory Standards Institute [24]. Commonly prescribed and commercially available antibiotics discs (Oxoid ltd) were used for antibiotic susceptibility testing. The antibiotics that were used with their concentration were quinolones- norfloxacin (5 μg) and ciprofloxacin (5 μg), aminoglycosides-gentamycin (10 μg), cephalosporins-cefoxitin (30μg), macrolides-azithromycin (15 μg) and Beta-lactam antibiotics- amoxillin clavulanic acid (30 μg) and ampicillin (20 μg) (Liofilm s.r.l Rosete (TE), Italy). A suspension of 0.5 McFarlands of bacterial isolate was prepared and cultured on Mueller-Hinton agar (MH) (Liofilm s.r.l Rosete (TE), Italy) by uniformly swabbing and spreading on MH solid agar media using a sterile swab. The antibiotics above were then placed aseptically on each cultured plate and incubated aerobically at 37^0^C for 18-24 hours. *Escherichia coli* ATCC 25922 was used as a control strain. Interpreting antibiotic susceptibility was done by measuring the zone of inhibition around each antibiotic using a ruler. The breakpoint for antimicrobial drugs was based on the guidelines provided by the Clinical and Laboratory Standards Institute [24]. This was used to determine the antibiotic susceptibility. The results were reported as susceptible, intermediate or resistant to a particular drug.

**Bias:** systematic random sampling methods were employed in selecting participants until the expected sample size was reached, to avoid bias. Sampling and subsequent analysis was also done three times in a week at an interval of one day for up to three months to avoid bias.

**Study size:** systematic random sampling technique was applied in this study. In this case, the participants were recruited in groups of five patients. A pilot study had showed that at least twenty-five patients visit the laboratory each with a request for the test on UTI per day. Since the research was carried out in a period of three months, the target sample population size was divided by the number of days in order to get the sample size per day. This was then divided by the approximated number of patients presenting UTI per day in order to get the interval at which systematic random sampling was done. The sample size was determined using a formula for estimating minimum sample size for prevalence studies [19].


$$n=\frac{z^{2}\times p\left(1-p\right)}{d^{2}}$$

where; n is the minimum sample size, p is the prevalence of UTI in the previous studies, which is 26.7% [20], z is the value corresponding to 95% confidence interval, and d is the significance level, 0.05.


$$n=\frac{1.96^{2}\times 0.267\left(0.733\right)}{0.05^{2}}$$

Therefore, 300 was the sample size required for the study.

**Statistical analysis:** coding, entry, cleaning and verification of data were using SPSS version 21 software. Descriptive statistics were used to analyse demographic characteristics and the rate of antibiotic resistance. Pearson correlation was used to test for association between organism and gender.

**Ethical and scientific approvals:** the study was done after obtaining authority from the Board of Graduate Studies of University of Kabianga. Ethical clearance was sought from the Ethical Committee of Kericho County Referral Hospital and the Institutional Research and Ethics Committee (IREC) of Moi Teaching and Referral Hospital, Eldoret.

## Results

A total of 300 samples were received for laboratory analysis for detection of urinary tract infection. Among these, 60 samples yielded bacteria isolates giving UTI prevalence of 20% (Table 1, Table 2). Of the total population, females consisted 67% and males 33%. Of those with confirmed with UTI, the majority were females (n=48; 80%), as compared to males (n=12; 20%) There were no participants with missing data.

**Table 1:** occurrence of UTI in the study population (N=300)

Study participants	Presence of UTI n (%)	Absence of UTI n (%)	Total participants N (%)
**Females**	48 (16)	153 (51)	201 (67)
**Males**	12 (4)	87 (29)	99 (33)
**Total**	60 (20)	240 (80)	300 (100)

**Table 2:** age-matched cases with urinary tract infection

Age	Females (n)	Males (n)	Total N (%)
1 to 10	1	0	1 (1.7)
11 to 20	3	1	4 (6.7)
21 to 30	27	7	34 (56.7)
31 to 40	8	1	9 (15)
41 to 50	4	1	5 (8.2)
51 to 55	2	1	3 (5)
61 and above	3	1	4 (6.7)
TOTALS	48	12	60 (100)

**Figure 1:**
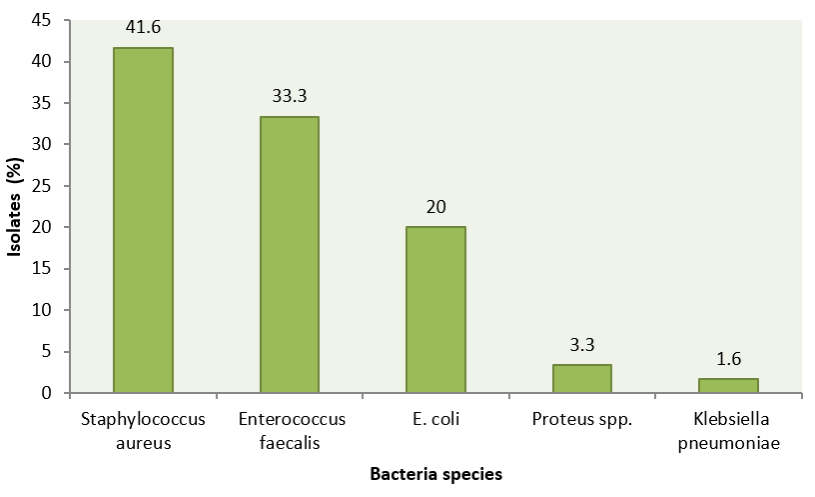
overall proportion of uropathogens isolated from patients with UTI

**Uropathogens identified in urine sample from patients with UTI:** eligible participants who consented to participate in the study were 300 hence 300 samples. Complete data were obtained from the samples and no participant had missing results. From the 300 samples received, 60 (20%) yielded bacterial isolates. Of the bacteria isolates detected, the majority were gram-positive cocci (GPC) which constituted 73% (n=45), while 27% (n=15) were gram-negative rods (Figure 1). *S. Aureus* (n=25; 41.7%) and *Enterococci faecalis* (n= 20; 33.3%) were the major aetiological agents, followed by *E. coli* (n=12; 20.0%), *Proteus* species (n=2; 3.3%) and *K. Pneumonia* (n=1; 1.6%). There was no statistically significant association between organisms causing UTI and gender (Pearson correlation=0.872).

**Figure 2:**
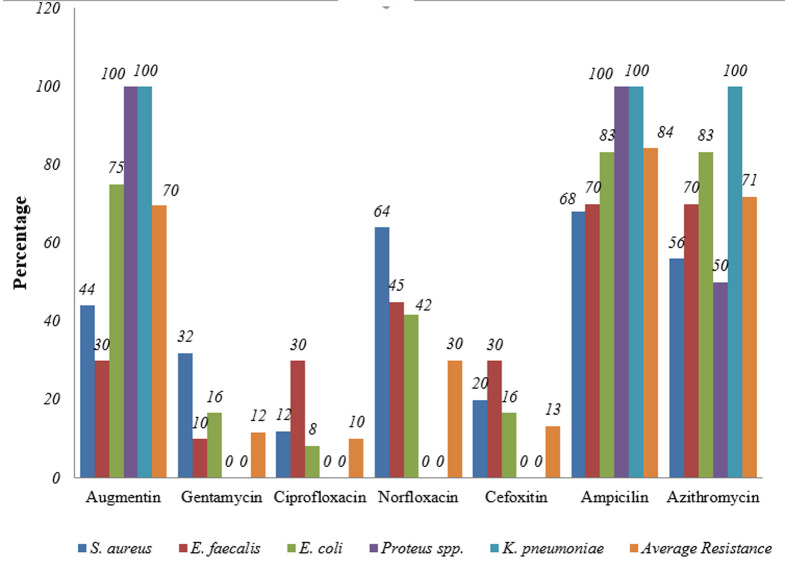
overall antibiotic resistance

**Antibiotic resistance profile of uropathogens isolated from patients with UTI:** bacteria isolates showed varying trends of susceptibility and resistance to the therapeutic agents. Overall, the respective antibiotic resistance patterns of uropathogens were as given in Table 3. *Staphylococcus spp, Enterococci spp* and *E. coli* showed at least resistance to all antibiotics tested in the study. Although *Klebsiella spp* and *Proteus spp* showed resistance to other antibiotics, no resistance to cefoxitine, gentamycin, ciprofloxacin and norfloxacin was observed as shown in Figure 2. Overally, uropathogens demonstrated high susceptibility to cefoxitine, gentamycin and ciprofloxacin. Augmentin and norfloxacin showed moderate efficacy, although norfloxacin showed slightly high resistance among gram positives. Significant antibiotic resistance among uropathogens was observed in augmentin, azithromycin and ampicillin. The commonly isolated aetiological agents showed multiple resistance to at most 5 drugs tested in the study (Table 3). Most of them were resistant to 3 drugs. *Enterococci* showed the highest resistance to most antibiotics, R5 (n=4; 20%), followed by *S. aureus* (n=3; 12%) and *E. coli* (n=1; 8.3%).

**Table 3:** bacteria resistance to antibiotics

Organism	Multiple resistance			
R2	R3	R4	R5
*S. aureus* (n=25)	4(16%)	11(44%)	3(12%)	3(12%)
*E. faecalis* (n=20)	6(30%)	4(20%)	1(5%)	4(20%)
*E. coli* (n=12)	3(25%)	4(33.3%)	4(33.3%)	1(8.3%)

R2=Resistance to two antibiotics; R3=Resistance to three antibiotics; R4=Resistance to four; R5=Resistance to five antibiotics

**Limitation:** the study was limited to the few antibiotics that were commonly prescribed for empirical treatment in Kericho County Referral Hospital.

## Discussion

Twenty percent (20%) isolation rate of bacteria showed that symptoms of UTI alone cannot be an adequate guide on which to base on diagnosis and subsequent antibiotic therapy in cases of urinary tract infection. This is because some sexually transmitted diseases are caused by organisms other than bacteria; protozoa, fungi and virus infections which mimic UTI. Interstitial cystitis, bladder cancer and prostate problems also present symptoms that are similar to UTI. Isolation rate obtained in this study compares closely to 20.6% obtained from a related study done in Kisii Teaching and Referral Hospital [25]. It therefore confirms that the low isolation rate cannot be due to improper sample handling. This finding is also in agreement with a study that was done in Tanzania which obtained prevalence of 23.3% [26] and in Ethiopia 14% [27]. The gram-positive bacteria *S. Aureus* which accounted for 41.7% was a predominant cause of community acquired UTI. In agreement to our study, research conducted by Kline and Lewis [5] found *Staphylococcus spp* as major causative agent of UTI accounting for 42%. Related studies conducted in other regions also found a higher prevalence of *E. coli* (61.3%) isolates from urine, others being *S. Aureus* (9.3%), *Enterobacter spp* (3.7%), *Klebsiella spp* (3.3%), *Citrobacter spp* (2.5%) and Proteus spp (2.1%) [28,29]. This can be explained by time and environmental variation, social habits of the community, the standard of personal hygiene and education. This indicates that variation in community-acquired UTI and antibiotic susceptibility should be done regularly in order to capture any emerging resistance. These organisms express a number of virulence factors which includes adhesion, motility, biofilm formation, immunoavoidance, and nutrient acquisition.

The various bacteria isolated in this study demonstrated varied resistance to antibiotics namely; ampicilin, azithromycin and augmentin. Considering the two groups of bacteria, Gram-positive cocci, *E. faecalis* and *S. Aureus* also demonstrated varied resistance to antibiotics. In this study, high resistance to norfloxacin and ampicillin was observed, confirming the earlier reported rates of these drugs of over 70% in Kenya, Ethiopia and Tanzania [20,26,30]. *Enterococci spp* showed high muti-drug resistance to most antibiotic agents. The ability of *Enterococci* to form biofilm on abiotic surfaces gives it important virulent and antibiotic resistance property [31]. Studies that have been conducted on *Enterococci* in relation to urinary tract infection demonstrated the bacteria to have *esp* gene, which is an important gene for biofilm formation [32]. These bacteria also exhibit decreased susceptibility to beta-lactam due to acquisition of penicillin binding protein (PBP) which hydrolyze beta-lactam antibiotics and is coded for by *pbp5* genes [33]. The acquisition and subsequent dissemination of these antibiotic resistance genes have led to spread of high-level ampicillin resistance clone of *Enterococci spp* which was also observed in this study.

The gram negative bacteria, *E. coli, Proteus spp* and *Klebsiella pneumonia* were highly resistant to ampicilin, azithromycin and augmentin, moderately resistant to norfloxacin and least resistant to cefoxitin, ciprofloxacin and gentamycin. This was in agreement with a study done in Kenya [25] who recorded antibiotic susceptibility profile of uropathogens to ampicillin (25%), nitrofurantoin (25%), cotrimoxazole (25%) and gentamycin (100%). A study conducted in Ethiopia [27] showed emerging uropathogenic *E. coli* resistant to ampicillin and augmentin. A similar study conducted in Rwanda demonstrated a higher proportion of *E. coli* strains (n= 119) which were resistant to amoxiclav (86%), nitrofurantoin (26.4%), nalidixic acid (45.1%) and gentamycin (36.1%), [34]. Compared to studies from other countries, the majority of antibacterials except third-generation cephalosporin, ciprofloxacin, cefepime, and cefoxitine were resistant [35]. World Health Organization National data from 5 regions reported at least 50% resistance to fluoroquinolones (ciprofloxacin, ofloxacin and norfloxacin) in *E. coli* and *Klebsiella spp* [36]. Gram-negative bacteria, the *Enterobacteriaceae* produce extended spectrum beta-lactamase (ESBL) enzyme that confer resistance to beta-lactam antibiotics. These bacteria were found to acquire beta-lactamase such as blaOXA-48 and blaCTX-M gene which hydrolysed penicilins and cefotaxime at high level but not expanded spectrum cephalosporins [37]. Thus, the high level of resistance of this clinical isolates to beta-lactams could be attributed to acquisition of peculiar beta-lactamases and modification of outer membrane proteins. To combat ESBL producing bacteria, cefoxitine which is a new, cephalosporin-like antibiotic and highly resistant to hydrolysis by β-lactamase have been used in the treatment. Only 10% showed resistance to cefoxitine showing that the drug may be considered an alternative for the treatment of resistant UTIs.

High resistance rate to antibiotics is a reflection of widespread use of these antibiotics combined with failure to follow ethics in antibiotic use, which vary from place to place [38]. The newer fluoroquinolones, namely ciprofloxacin and norfloxacin showed good efficacy but decreasing in vitro efficacy for the isolates with resistance rates of 20%. Gentamycin, an aminoglycoside showed high efficacy for all isolates (78%). This drug has been in use for over 50 years and still retains its potency. Gentamycin has a limited use, probably because of the injection route of administration, which is not desirable to many patients. This limited use has delayed the emergence and spread of resistance to the drug. Review of other literature in Kenya and other regions of the world revealed that antibiotic resistance is normally higher in cases where there is high usage of antibiotics hence directly associated with misuse of antibiotics [39,40] Continuous development of antibiotic resistance in currently susceptible population suggests that some bacteria mutate to the resistant state. This study confirms an increasing prevalence of infection caused by antibiotic-resistant bacteria, which complicates the empirical treatment of UTI. Bacterial resistance to commonly used antibiotic is worrying, especially the resistance to the few remaining effective antibiotics [41]. With paucity in the development of new antibiotics, the world is heading to an era where diseases that were once treatable would potentially be deadly.

**Funding:** this research did not receive any funding from any institution or organization.

## Conclusion

While, clinical picture should be put into perspective and laboratory evaluation done before treatment of UTI. The study confirmed the existence of antibiotic resistance, both single and multiple, in the various bacteria causing UTI. It thus, contributes towards data on current antimicrobial resistance in Kenya. The study recommends antibiotic susceptibility tests to be done regularly in order to capture any emerging resistance. A surveillance system should therefore be put in place to monitor the trends and emergence of antimicrobial resistance. This will ensure continuous research into its prevention and will aid the policymakers in the fight towards reducing antimicrobial resistance. The study also recommends more studies to be done in other regions of Kenya using more antimicrobials to get a diverse antimicrobial susceptibility that will aid in treatment of UTI.

### What is known about this topic

- *Urinary tract infection is common in females than in males;*
- *E. coli is the predominant aetiological agents;*
- *Antibiotic resistance exists among E. colistrains*.

### What this study adds

- *This study found another predominant aetiological agent;*
- *Resistance to at most 5 drugs showed increasing level of antibiotic resistance and the need for regular antibiotic susceptibility screening*.
